# Autologous Fat Grafting for Post-mastectomy Pain Syndrome: A Systematic Review and Meta-Analysis

**DOI:** 10.7759/cureus.49017

**Published:** 2023-11-18

**Authors:** Sabrina Samuels, Teniola Adeboye, Abdal Qadir Zafar, Chie Katsura, Charlie Izard, Nazanin Shahrokhi, Shafiq Rahman

**Affiliations:** 1 Plastic and Reconstructive Surgery, Sheffield Teaching Hospitals NHS Foundation Trust, Sheffield, GBR; 2 Plastic and Reconstructive Surgery, Mid and South Essex NHS Foundation Trust, Essex, GBR; 3 Orthopaedic Surgery, Leeds Teaching Hospitals NHS Trust, Leeds, GBR; 4 Plastic and Reconstructive Surgery, Hull University Teaching Hospitals NHS Trust, Hull, GBR; 5 Plastic and Reconstructive Surgery, Leeds Teaching Hospitals NHS Trust, Leeds, GBR; 6 Plastic and Reconstructive Surgery, Manchester University NHS Foundation Trust, Manchester, GBR

**Keywords:** systematic review and meta-analysis, post-mastectomy complications, management of pmps, neuropathic pain, chronic pain management, post-mastectomy pain syndrome

## Abstract

Fat grafting has been described as a potential treatment for post-mastectomy pain syndrome (PMPS) following oncological breast surgery. The study's aim was to compare and contrast the current literature using a systematic review and meta-analysis to quantify the evidence.

The Preferred Reporting Items for Systematic Reviews and Meta-Analyses (PRISMA) guidelines were used. Databases, including MEDLINE, Google Scholar, Cumulative Index to Nursing and Allied Health Literature (CINAHL), and the Cochrane Central Register of Controlled Trials (CENTRAL), were searched. Data synthesis was conducted using Review Manager 5.4 (Cochrane Collaboration, London, UK), with 95% confidence intervals. All randomised controlled trials (RCT) and observational studies comparing lipofilling for PMPS were included. A total of six studies met the inclusion criteria with five articles being used in data analysis for the mean percentage reduction in visual analogue scale (VAS) score. The primary outcome measure was the mean percentage reduction in the VAS pain score. Secondary outcomes included the Neuropathic Pain Symptom Inventory (NPSI) and the quality of life assessments post treatment.

Overall, a total of 266 patients received fat transfer for PMPS, and 164 were in the control group. The mean percentage reduction in VAS score was 19.8 (10.82, 28.82; p < 0.0001). Secondary outcomes, including health-related quality of life, showed good outcomes post fat transfer. This involved breast softness, cosmesis, and psychosocial well-being.

The results from this meta-analysis suggest that autologous fat grafting is an efficacious treatment for reducing pain caused by PMPS. The authors suggest more high-quality trials are needed to enhance the current evidence base.

## Introduction and background

Post-mastectomy pain syndrome (PMPS) is a common sequela in oncological breast surgery. It is defined by the International Association for the Study of Pain as chronic pain in the anterior thorax, the axilla, and or the upper half of the arm, lasting more than three months after lumpectomy or mastectomy [1]. It is typically neuropathic in nature, described as a dull, burning, and aching sensation exacerbated by movement of the shoulder girdle [2]. Fat grafting has been described as a method of reducing PMPS. Caviggioli et al. showed a significant decrease in patients with PMPS treated with autologous fat tissue grafting [3]. The mechanism of PMPS is unclear but thought to be related to the release of tissue with the liberation of entrapped nerves [2]. Others have suggested that mesenchymal stem cells have immunosuppressive effects that inhibit the proliferation of CD4+ and CD8+ T lymphocytes, leading to an analgesic effect from inflammation inhibition [4].

To the authors' knowledge, the literature is devoid of a systematic review and meta-analysis to quantify the outcomes of studies assessing the use of autologous fat grafting for the treatment of PMPS. This study is the first to report on the topic and amalgamate the evidence.

## Review

A systematic review and meta-analysis were performed as per the Preferred Reporting Items for Systematic Reviews and Meta-Analyses (PRISMA) guidelines [5].

Eligibility criteria

All prospective randomised and non-randomised control trials as well as observational studies comparing lipofilling of patients following oncological surgery for breast cancer with a control group were included. There was no differentiation between mastectomies or wide local excisions. Both were considered as part of this review. Only patients with a diagnosis of PMPS were recruited. Inclusion was not restricted by age, sex, comorbidity status, or receipt of adjuvant or neoadjuvant surgery, including ancillary surgery or the type of reconstruction. Case series and case reports were excluded, as well as articles not reported in English.

Primary and secondary outcomes

The primary outcome was the mean percentage reduction in the visual analogue scale (VAS) pain score. Secondary outcomes included the degree of neuropathic pain using the Neuropathic Pain Symptom Inventory (NPSI) as well as quality-of-life assessments post treatment.

Literature search strategy

Two authors independently searched the following electronic databases: MEDLINE, Google Scholar, Cumulative Index to Nursing and Allied Health Literature (CINAHL), and the Cochrane Central Register of Controlled Trials (CENTRAL). The search was run on 15 April 2023. No language restrictions were applied in our search strategies. The search terminologies included ‘fat transfer’, ‘fat graft’, ‘post-mastectomy pain syndrome’, ‘PMPS’, ‘oncological excisions’, ‘wide local excision’, ‘breast conserving surgery’, and ‘mastectomy’. These search terms were combined with adjuncts of ‘AND’ or ‘OR’.

Selection of studies

Each author independently assessed the title and abstract of all articles identified from the literature search. The full texts of relevant reports were retrieved and those articles that met the eligibility criteria of our review were selected. Discrepancies in the study selection between the authors were resolved by discussion with another co-author.

Data extraction and management

An electronic data extraction spreadsheet was created in line with Cochrane’s data collection form for intervention reviews. The spreadsheet was pilot-tested in randomly selected articles and adjusted accordingly. Our data extraction spreadsheet included study-related data (first author, year of publication, study design, number of patients in the treatment and control groups), baseline demographics of the included populations (age, BMI, ethnicity), and initial operative details (axillary dissection, adjuvant treatments, type of oncological surgery, reconstruction).

Data synthesis

Review Manager 5.4 software (Cochrane Collaboration, London, UK) was used to conduct data synthesis. The extracted data were entered into Review Manager by two authors independently (A.Q.Z and C.I.). A random effects model was used for analysis. The results were reported in forest plots with 95% confidence intervals (CIs). For continuous outcome data, the mean difference (MD) was used to assess both groups.

Assessment of heterogeneity

Heterogeneity among the studies was assessed using Cochrane's Q test as well as calculating the I2 score, which was interpreted using the following scale: 0-25% = low heterogeneity; 25-75% = moderate heterogeneity; and 75-100% = high heterogeneity.

Results

**Figure 1:**
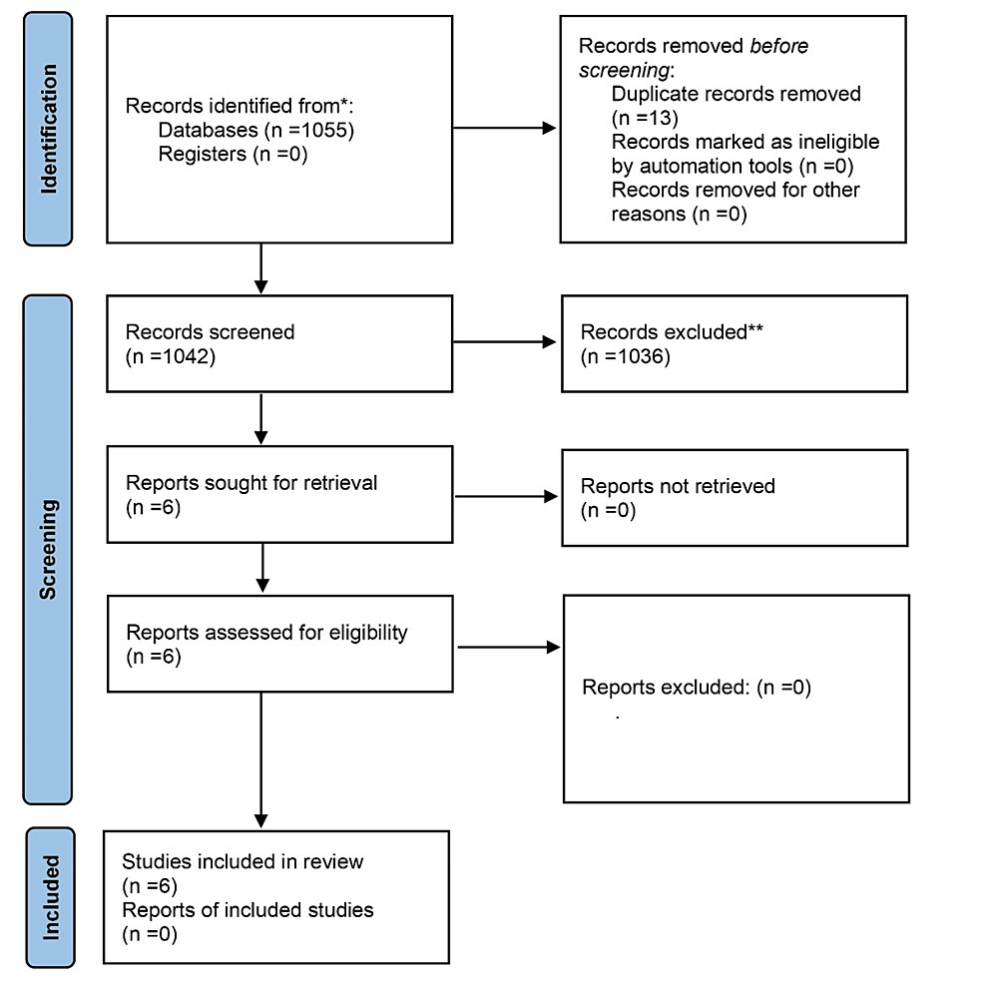
PRISMA flowchart for article screening and selection for fat grafting in post-mastectomy pain syndrome PRISMA: Preferred Reporting Items for Systematic Reviews and Meta-Analyses.

The search strategy retrieved 1055 studies in total (Figure 1). Of these, six studies were identified that met the eligibility criteria, after meticulous screening by three independent reviewers.

Description of studies

Caviggioli et al. (2011)

A single-centre, prospective, non-randomised control study of 113 patients, who suffered from PMPS and severe scar retractions after unilateral mastectomy with axillary dissection, radiotherapy, and implant breast reconstruction. The intervention group underwent autologous fat grafting to the breast, while the control group did not receive any intervention [3].

Maione et al. (2014)

A single-centre, prospective, non-randomised control study of 96 patients, who suffered from PMPS and severe scar retractions after unilateral lumpectomy and radiotherapy. The intervention group underwent autologous fat grafting to the breast, while the control group did not receive any intervention [6].

Juhl et al. (2016)

A single-centre, prospective, non-blinded, randomised control study of 18 patients, who developed persistent pain after breast cancer therapy (PPBCT) after unilateral mastectomy with or without radiotherapy and did not have breast reconstruction. The intervention group underwent autologous fat grafting while the control group did not receive any intervention [7].

Caviggioli et al. (2016)

A single-centre, prospective, non-randomised control study of 209 patients, who suffered from PMPS mastectomy with axillary dissection or quadrantectomy. All patients received radiotherapy post oncological surgery. The interventional group underwent autologous fat grafting to the breast, while the control group did not receive intervention [8].

Cogliandro et al. (2017)

A single-centre, prospective, non-randomised control study evaluating patient satisfaction post-operatively using Breast-Q reconstruction module among 70 patients, who had a mastectomy with definitive implant breast reconstruction. This study compared Breast-Q outcomes among patients, who did and did not receive subsequent lipofilling after implant reconstruction [9].

Sollie et al. (2022)

A single-centre, double-blinded, randomised control study of 35 patients, who suffered from PMPS without breast reconstruction. The intervention group underwent scar-releasing rigottomy, liposuction, and autologous fat grafting to the breast. The control group underwent liposuction, scar-releasing rigottomy, and sham injection of saline to the breast [10].

An amalgamated table was created to include studies comparing the use of fat grafting versus no fat grafting for PMPS (Table 1). The following baseline characteristics were included in the table: study design, number of patients, age, BMI, axillary dissection, adjuvant treatment, method of fat harvest, type of oncological surgery, and reconstruction.

**Table 1:** Amalgamated results for included studies comparing the use of fat grafting versus no fat grafting for post-mastectomy pain syndrome BMI: body mass index; SD: standard deviation; NR: not reported; TE: tissue expander; NAC: nipple-areola complex.

First author and year	Design	No. of patients		Age (years)		BMI (kg/m^2^)		Axillary dissection		Adjuvant treatment		Method of fat harvest	Type of oncological surgery	Reconstruction
		Fat graft	Control	Fat graft	Control	Fat graft	Control	Fat graft	Control	Fat graft	Control			
Sollie et al. (2022) [10]	Single-centre, double-blind, randomised controlled trial	18	17	Mean (SD) age: 63.8 (9.9)	Mean (SD) age: 61.0 (8.8)	Mean (SD) BMI: 26.3 (3.5)	Mean (SD) BMI: 26.2 (3.7)	12	11	Chemotherapy: 14; radiotherapy: 18; anti-oestrogen therapy: 16	Chemotherapy: 10; radiotherapy: 13; anti-oestrogen therapy: 17	Coleman’s technique	Mastectomy	NR
Cogliandro et al. (2017) [9]	Prospective cohort study	46	24	Mean age: 41		NR		NR		Radiotherapy: 34; chemotherapy: 39	Radiotherapy: 13; chemotherapy: 16	Coleman’s technique	Mastectomy	Implant
Juhl et al. (2016) [7]	Randomised controlled trial	8	7	Mean ± SD (range) age: 59.9 ± 9.8 (49.3-74.3)	Mean ± SD (range) age: 58.9 ± 7.4 (50.2-69.4)	Mean ± SD (range) BMI: 26.0 ± 6.5 (18.4- 34.3)	Mean ± SD (range) BMI: 23.7 ± 5.4 (17.7- 33.4)	6	5	Chemotherapy: 6; radiotherapy: 6; anti-oestrogen therapy: 6	Chemotherapy: 4; radiotherapy: 4; anti-oestrogen therapy: 7	Coleman’s technique	Mastectomy	None
Caviggioli et al. (2016) [8]	Prospective cohort study	131	78	NR		NR		113 intervention vs. control not reported		All subjects underwent adjuvant radiotherapy		Coleman’s technique	Mastectomy vs. quadrantectomy	NR
Maione et al. (2014) [6]	Single-centre case-control	59	37	Mean (range) age: 51 (33-68)	Mean (range) age: 54 (36-67)	Mean (range) BMI: 30.4 (22.5- 38.6)	Mean (range) BMI: 29.5 (21.4-39.0)	38	23	All subjects underwent adjuvant radiotherapy		Coleman’s technique	Lumpectomy	NR
Caviggioli et al. (2011) [3]	Case-control study	72	41	NR		NR		All subjects had axillary dissection		All subjects underwent adjuvant radiotherapy		Coleman’s technique	Mastectomy	Subpectoral TE was subsequently replaced with an implant; separate reconstruction of NAC 12 months later

**Figure 2:**
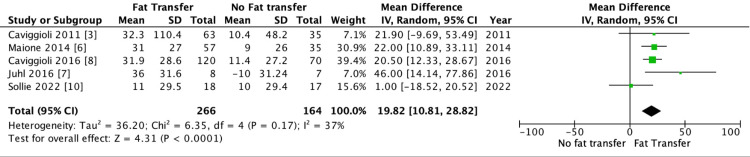
Mean difference analysis to assess the average percentage in pain reduction scores for post-mastectomy pain syndrome using fat transfer

The forest plot below demonstrates a significant result for the mean percentage pain reduction using the VAS score for fat transfer when treating PMSP (p < 0.05). The mean difference is 19.82 (10.81, 28.82) (Figure 2).

Assessment tools for included studies

The Newcastle-Ottawa quality assessment tool was used to assess the quality of the included observational studies (Table 2) [11].

**Table 2:** Newcastle-Ottawa Scale assessment Selection: maximum five stars; comparability: maximum two stars; outcome: maximum three stars.

Newcastle-Ottawa Scale assessment			
	Selection	Comparability	Outcome
Caviggioli et al. (2011) [3]	****	**	**
Maione et al. (2014) [6]	****	**	**
Caviggioli et al. (2016) [8]	****	**	**
Cogliandro et al. (2017) [9]	****	*	**

The Cochrane Collaboration Tool was used to assess the risk of bias in randomised controlled trials (RCTs) (Table 3).

**Table 3:** A comparison of RCT assessment of bias RCT: randomised controlled trial.

Cochrane Collaboration Tool for RCTs			
	Bias	Authors’ judgement	Support for judgement
Juhl et al. (2016) [7]	Random sequence generation (selection bias)	Low risk	Randomised with permuted block design
	Allocation concealment	High risk	No information of concealment
	Blinding of participants and personnel (performance bias)	High risk	This study was not blinded
	Incomplete outcome data (attrition bias)	High risk	The patient was not blinded; the assessment was based on the patient's self-reporting
	Incomplete outcome data (attrition bias)	Low risk	A small number of attritions and reasons were clearly stated
	Selective reporting (reporting bias)	Low risk	Pre-defined outcome as per study protocol
	Other bias	Low risk	Similar baseline characteristics
Sollie et al. (2022) [10]	Random sequence generation (selection bias)	Low risk	Patients were randomised using a computer algorithm
	Allocation concealment	Low risk	Allocation mid-surgery
	Blinding of participants and personnel (performance bias)	Low risk	Patients were informed of allocation only at follow-up
	Incomplete outcome data (attrition bias)	Low risk	Performing surgeons were not involved in follow-up and assessment
	Incomplete outcome data (attrition bias)	Low risk	A small number of attritions and reasons were clearly stated
	Selective reporting (reporting bias)	Low risk	Pre-defined outcome as per study protocol
	Other bias	Low risk	Similar baseline characteristics

Discussion

The authors report the first meta-analysis within the literature to assess the effects of fat grafting in the treatment of PMPS. A significant percentage reduction was evidenced in the VAS pain scores (p < 0.05) utilising a mean difference analysis. Secondary outcome measures including NPSI, which is an assessment tool for neuropathic pain, showed significant improvement in pain, as reported by Juhl et al., but Sollie et al. showed no differences between control and intervention groups for fat transfer when treating PMPS [7,10]. Juhl et al. also assessed pain intensity and health-related quality of life (HRQoL), both of which demonstrated an improvement [7]. In contrast, Sollie et al. showed no significant differences in quality of life using the 36-item Short Form Health Survey [10]. The only reported item demonstrating significant change was role limitations due to emotional problems. Cogliandro et al. used the Breast Q survey to assess patient satisfaction and significant differences were evidenced in the lipo-transfer group with an improved appearance in clothing, breast softness, cosmesis, as well as psychosocial well-being [9]. In addition, general pains in the breasts were reported to be much improved subsequent to fat grafts. Juhl et al. demonstrated better scar quality overall using the Patient and Observer Scar Assessment Scale (POSAS) observer tool for all subscales except vascularity and in the patient-reported scores, colour as well as stiffness was improved [7].

The exact pathophysiology of PMPS remains unclear; however, several theories have been hypothesised. The process of fibrosis has been identified as a possible cause for PMPS. Scar tissue formed post deep dissection may cause the entrapment of nerves, which are consequently excited by post-operative seromas, hematomas, or infections [12,13].

Adipose stem cells, anti-inflammatory molecules, and growth factors have all been shown, in vivo, to alter neuropathic hypersensitivity [14]. This blunts the pain response and has the potential to reinnervate the skin [15-17]. The multipotency of these adipose cells has also been shown in in-vivo studies to increase scar softness using fat. The main postulated theory is via stem connective tissue regeneration, thus releasing nerve pressure [18-20].

Oncological breast surgery is a traumatic process, which upregulates stress and inflammatory responses. Post-traumatic scars have intrinsic inhibitory factors, which are expressed by the extracellular matrix [21]. Maione et al. hypothesised that adipose stem cells modulate the hostile microenvironment of post-traumatic scars through the induction of molecular changes [6].

Adipose stem cells also have the potential to downregulate the immune response by reducing T-cell activation, reducing the production of pro-inflammatory cytokines, limiting B-cell terminal differentiation, and inhibiting natural killer (NK) cell proliferation. Their impact on paracrine immunosuppression by soluble growth factors is also present [6,22].

Overall, the quantitative assessment of this study showed significant improvement in pain with fat transfer as measured by the mean percentage reduction in VAS scores with moderate reported overall heterogeneity giving further consistency to the quantitative outcome. Neuropathic pain can be difficult to treat and often a combination of different modalities appears to be more effective [23,24]. Pharmacological treatment has often been the mainstay but with the emerging evidence of fat transfer, it offers a promising adjunct to managing PMPS [6].

The reported outcomes of the current review should be studied in the context of inherent limitations. Not all studies were RCTs and the included observational studies reported poor scores for compatibility on the Newcastle-Ottawa assessment although they scored well for the selection and exposure domains. The Cochrane Collaboration tool assessment for the RCT was graded as low risk for the majority of different bias domains. However, heterogeneity was moderate across all studies giving further consistency to the quantitative outcome. The study follow-up periods were at baseline, three, and six months for the RCT and for the observational studies, follow-up ranged, on average, from 10 months to two and a half years.

This meta-analysis has shown the beneficial effects of fat transfer in managing PMPS following oncological breast surgery. However, more high-quality RCTs with larger sample sizes will be needed to enhance the current evidence base. In addition, better subgroup analysis is needed to account for confounding factors, including radiotherapy and chemotherapy, as well as those who have had ancillary procedures, including axillary clearances to exclude potential sources of bias.

## Conclusions

The results of this meta-analysis suggest that fat grafting is a useful treatment option for reducing chronic pain associated with PMPS. This is a novel form of management for PMPS and should be offered to patients during the onset of symptoms and strongly considered where other treatment methods have failed. The current review is limited by the small number of studies and the authors suggest more randomised trials are needed to enhance the evidence.
